# Prevalence, predictive factors, and the influential role of physical activity trajectories of cancer-related cognitive impairment among patients newly diagnosed with non-small cell lung cancer

**DOI:** 10.1007/s00520-026-11368-5

**Published:** 2026-10-24

**Authors:** Mu-Hsing Ho, Janette L. Vardy, Yen-Kuang Lin, Wing Lok Chan, Naomi Takemura, Lizhen Wang, Denise Shuk Ting Cheung, Chia-Chin Lin

**Affiliations:** 1 School of Nursing, Li Ka Shing Faculty of Medicine, The University of Hong Kong, Pokfulam, Hong Kong SAR; 2 Australian Research Centre for Cancer Survivorship, Faculty of Medicine and Health, University of New South Wales, Sydney, NSW Australia; 3 Graduate Institute of Athletics and Coaching Science, National Taiwan Sport University, Taoyuan, Taiwan; 4 Department of Clinical Oncology, Li Ka Shing Faculty of Medicine, The University of Hong Kong, Pokfulam, Hong Kong SAR; 5 School of Nursing, Faculty of Health and Social Sciences, The Hong Kong Polytechnic University, Hung Hom, Hong Kong SAR; 6 Alice Ho Miu Ling Nethersole Charity Foundation Professor in Nursing, Pokfulam, Hong Kong SAR

**Keywords:** Cancer, Cancer-related cognitive impairment, Group-based trajectory modelling, Non-small cell lung cancer, Physical activity trajectories

## Abstract

**Purpose:**

Evidence on the prevalence, trajectory, and predictors of cancer-related cognitive impairment (CRCI) in newly diagnosed non-small cell lung cancer (NSCLC) patients remains limited, particularly regarding the role of physical activity patterns. This study aimed to estimate the prevalence of CRCI, assess changes in objective and subjective cognitive functions, and identify predictors of CRCI, focusing on physical activity trajectories among patients newly diagnosed with NSCLC in Hong Kong.

**Methods:**

A prospective observational study was conducted with 327 NSCLC patients newly diagnosed within 4 weeks. Cognitive function was objectively assessed using the HK-MoCA 5-Minute Protocol and subjectively measured using the FACT-Cog scale at baseline and monthly for six months. Physical activity patterns were analyzed through group-based trajectory modelling. Multivariate logistic regression identified predictors of CRCI, adjusting for demographic and clinical factors.

**Results:**

The prevalence of objective CRCI ranged from 17.5% to 29.1% over the 6-month period, with 24.1% affected at 6 months. Three distinct physical activity trajectories emerged: consistently low (43.1%), consistently high (11.9%), and declining from high to moderate activity (45%). Both consistently high and declining physical activity trajectories were associated with a significantly reduced CRCI risk (93.8% and 85.9% lower risk, respectively) compared to the consistently low activity group. Consistently high physical activity trajectories were also correlated with better objective and subjective cognitive performance. Additional predictors of CRCI included lower baseline perceived cognitive function and self-efficacy for exercise.

**Conclusion:**

This study provides novel evidence that physical activity trajectories are associated with CRCI in newly diagnosed lung cancer patients.

**Supplementary Information:**

The online version contains supplementary material available at https://doi.org/10.1007/s00520-026-11368-5.

## Introduction

Lung cancer is the most common cancer and the leading cause of cancer-related deaths globally, with 80 to 85% being non-small cell lung cancer (NSCLC) [1, 2]. For advanced or inoperable cases, chemotherapy, radiotherapy, or combined treatments are primary options, yet survival rates remain lower than for other common cancers [3]. While cancer-related symptoms are well-established risk factors for survival and quality of life [2], cognitive impairment in lung cancer patients is understudied, with limited research on its mechanisms, prevalence, predictors, and management [4].

Cancer-related cognitive impairment (CRCI) describes the difficulties in thinking and memory that many patients with cancer experience, including issues with short-term memory, attention, executive function, language understanding, orientation, and how information is processed. These cognitive problems can arise as a result of cancer itself or its treatments and often include trouble forming thoughts, word-finding, multitasking, organizing, and completing familiar tasks due to mental slowing or decreased concentration [4]. Over half of individuals diagnosed with breast cancer or other cancers outside of the central nervous system experience CRCI following chemotherapy [4], while a proportional meta-analysis in lung cancer patients reported a pooled prevalence of 26% for CRCI [5]. The International Cancer and Cognition Task Force (ICCTF) highlights the likely effects of cancer treatments and pathophysiological mechanisms on the development of CRCI [6]. CRCI often diminishes self-confidence, affects social relationships, and makes daily tasks and work performance more challenging [4, 7].

Subjective cognitive function is also considered an important subdomain of CRCI, although research has shown a discrepancy or no relationship between subjective and objective cognitive function [16]. Studies that adopt appropriate self-reported CRCI measurement tools as recommended by the Cancer Neuroscience Initiative Working Group are required [8], such as the Functional Assessment of Cancer Therapy–Cognitive Function (FACT-Cog) or PROMIS Cognitive Abilities and Cognitive Concerns Scales. Cognitive function is complex and worth further investigation among lung cancer patients with both objective and perceived observations. Thus, including a measure to investigate subjective cognitive function has been considered as an essential aspect of CRCI measurement [8]. A comprehensive network meta-analysis has shown the traditional Chinese medicine-based interventions such as Tai Chi was effective in improving subjective cognitive complaints in cancer patients [9]. However, the heterogeneity between the included intervention studies was large. Despite some intervention studies having been implemented, the optimal strategy to combine those interventions has not yet been determined. Thus, identifying the predictors of CRCI and how physical activity plays a role in improving CRCI is essential and can contribute to future interventions that are tailor-made for the prevention of CRCI [10].

Key risk factors for CRCI include older age, pre-existing cognitive issues, cancer treatments (i.e., chemotherapy, surgery, or radiation), cancer symptoms, and physical activity levels [11, 12]. An updated review summarized how exercise and physical activity affect CRCI, showing that greater levels of physical activity and participation in exercise interventions are likely to help prevent or reduce cognitive problems in people with cancer [13]. There is evidence that exercise and physical activity can alleviate CRCI in breast cancers and other non-CNS cancers [12–15]. An integrated review found that exercise may be a feasible intervention to reduce cognitive decline [16]. However, the timing for implementing exercise programmes that increase physical activity remains unknown. Further, most of the studies were focused on patients with breast cancer or non-CNS cancer in terms of the prevalence and risk factors of CRCI [17, 18]. There is limited high-quality evidence investigating CRCI among lung cancer patients, yet an increased incidence of lung cancer has resulted in larger numbers of cancer patients struggling with this neurotoxicity [1, 19]. Therefore, evidence regarding prevalence and predictors of CRCI in lung cancer patients are needed to underpin the clinical recognition of CRCI.

Lung cancer patients who develop CRCI have their quality of life affected and likely diminished functional independence. Limited research has been undertaken on CRCI in patients newly diagnosed with lung cancer. This information is of fundamental importance regarding the epidemiology of lung cancer prognosis. The most sensitive period that the patient is at risk of developing CRCI after receiving a lung cancer diagnosis is unknown. Therefore, this study aimed to (1) estimate the prevalence of CRCI, (2) assess changes in objective and subjective cognitive function and their subdomains, and (3) identify the predictors of CRCI in patients newly diagnosed with NSCLC. We proposed the following research hypothesis: patients with a trajectory of decline in physical activity are more likely to develop CRCI.

## Methods

### Study design

This prospective, observational cohort study followed the Strengthening the Reporting of Observational Studies in Epidemiology (STROBE) reporting guidelines for cohort studies [20]. The outcome measures were conducted at baseline and monthly follow-ups until the 6th month after the recruitment. A 6-month observation period with monthly follow-ups was determined because the 6-month prognosis of patients with newly diagnosed lung cancer has been commonly adopted and considered in cancer care research to understand the long-term effects of the lung cancer diagnosis [21]. The timeframe with a 6-month observation period provided a context appropriate “cancer prognosis” for the follow-up measurements.

### Participants and setting

The inclusion criteria of the participants were patients who were (1) aged ≥ 18 years; (2) newly diagnosed with NSCLC (within 4 weeks); and (3) able to read and communicate in Cantonese, Mandarin or English. Patients diagnosed with cognitive impairment or dementia prior to the commencement of the study were excluded. We used apparent prevalence with specified precision to estimate the required number of subjects. With the following parameters: estimated true proportion = 0.75 (75% of the CRCI prevalence rate in cancer patients during treatment); desired precision ( ±) = 0.05; confidence level = 0.95; population size for finite populations = 35,082 (according to the new cancer cases diagnosed in Hong Kong in 2019 [19]). The result shows that 287 patients were considered sufficient. To take attrition into consideration, according to our previous work [22], targeted sample size was 319 patients allowing for a 10% attrition rate.

Convenience sampling was used to select participants who were easily accessible at the research site. Trained research assistants approached individuals at the location and explained the purpose and procedures of the study. Patients were recruited consecutively from the Department of Clinical Oncology and the Department of Medical Oncology at Queen Mary Hospital and Gleneagles Hospital in Hong Kong. The study sites included the clinical oncology clinics and departments where cancer cases are scheduled for diagnostic disclosure, treatment, and survivorship care. In addition, recruitment posters were displayed at several community sites including District Health Centres (DHC), Neighbourhood Elderly Centres (NEC), and District Elderly Community Centres (DECC). Potential participants who were interested were telephone-screened and eligible participants were scheduled for an in-person visit for the baseline measurement. Patients who agreed to participate in this study were asked to complete written consent and to complete the baseline survey immediately.

### Measurements

The survey has been piloted for readability and understandability and all measures used have been previously validated and tested in the lung cancer population. The whole survey takes approximately 25–30 min to complete. The battery of measurement tools designed to assess CRCI and its related predictors was selected following a review of the literature. Seven data collection time points, including a baseline and 6 monthly follow-ups, were implemented. All follow-up assessments were conducted in face-to-face interviews by trained research assistants. A 6-month observation period with monthly follow-ups was selected because the 6-month prognosis of patients with newly diagnosed lung cancer has been commonly adopted and considered in cancer care research to understand the long-term effects following a lung cancer diagnosis [13, 15]. The timeframe with a 6-month observation period provided a context appropriate cancer prognosis for the follow-up measurements. Data were collected at baseline with monthly follow-ups until the 6th month (T1 to T6).

### Dependent variable: cancer-related cognitive impairment

Objective cognitive function was assessed by the HK-MoCA 5-MIN (Hong Kong Montreal Cognitive Assessment 5-min) Protocol version. Objective CRCI was operationalized as an HK-MoCA 5-MIN total score at or below the seventh percentile of age- and education-adjusted normative data. The HK-MoCA 5-MIN includes four subtests examining cognitive subdomains, namely memory, language, orientation, and delayed memory. This measure has excellent test–retest reliability, and is highly correlated with the original MoCA [23].

### Independent variable: physical activity trajectory

Physical activity (PA) was measured by the IPAQ-SF (International Physical Activity Questionnaire- Short Form) that includes 7 items asking about “the time you spent being physically active in the last 7 days” to the respondents. The total physical activity in MET-minutes per week was calculated by summing time spent across walking, moderate, and vigorous activities weighted by their MET values. The physical activity levels were classified into three categories: “Low,” “Moderate,” and “High” [24].

### Control variables

#### Demographic characteristics

Demographic characteristics and cancer-related variables were collected including age, sex, duration of education in years, cancer stage, and treatments scheduled such as surgery, chemotherapy, radiation therapy, immunotherapy, and targeted therapy. Treatment information was collected at baseline only. Subsequent treatment initiation, changes in treatment modality, and treatment completion status were not assessed.

#### Subjective cognitive function

Subjective cognitive function was assessed using the 37-item FACT-Cog (Functional Assessment of Cancer Therapy–Cognitive Function) scale. FACT-Cog is designed for assessing perceived cognitive dysfunction and is validated in cancer patients. Four subdomains, including perceived cognitive impairments (CogPCI), impact of perceived cognitive impairments on quality of life (CogQOL); comments from others (CogOth), and perceived cognitive abilities (CogPCA) were calculated. This tool is recommended by the Cancer Neuroscience Initiative Working Group for measuring self-reported cognitive function [8].

#### Self-efficacy for exercise

The self-efficacy for exercise was evaluated by the SEE (Self-Efficacy for Exercise) scale, asking “how confident are you right now that you could exercise three times per week for 20 min if…” and followed by 9 items on a 10-point Likert scale. High internal consistency (alpha = 0.92), content validity and construct validity of the SEE have been confirmed [25].

#### Cancer symptom severity and interference

Cancer-related symptoms were monitored by the 19-item MDASI (The MD Anderson Symptom Inventory) which includes pain, exhaustion, nausea, disrupted sleep, distress/feeling upset, shortness of breath, difficulty remembering, loss of appetite, sleepiness, dry mouth, depression, vomiting, and numbness/tingling. Walking, activity, working (including housework), relations with other people, enjoyment of life, and mood are also included in the assessment as MDASI interference items [26]. The content validity and contrast validity of the traditional Chinese version measurement has been confirmed [27].

### Ethical considerations

This study was conducted in accordance with the ethical principles outlined in the Declaration of Helsinki. The research protocol underwent review and received approval from the Institutional Review Board of the University of Hong Kong and the Hospital Authority Hong Kong West Cluster (IRB/REC Ref. No.: UW 22-732) and the University of Hong Kong–Gleneagles Hospital Hong Kong Institutional Review Board (HKU-GHK IRB Ref. No.: GHK-2022-S007).

### Statistics analysis

Two-sided hypothesis tests were employed for all analyses, with the level of statistical significance set at α= 0.05. Group differences were quantified using the standardized mean difference. For continuous variables, independent samples *t*-tests were utilized, while categorical variables were examined using the chi-squared *χ*^2^ test.

Group-based trajectory modelling (GBTM) was applied to analyze patterns of physical activity and to classify participants into distinct trajectory groups. The method assigns individuals to clusters characterized by comparable trajectories, using each person’s repeated measures and modelling these paths with polynomial functions of time. This allows for individual differences in activity changes to be captured and summarized by distinct trajectory patterns [28]. The Bayesian Information Criterion (BIC) was utilized to evaluate the model fit of the GBTM results. After identifying distinct physical activity trajectories, logistic regression analysis was conducted to assess factors significantly predicting CRCI. Participants who died during follow-up contributed their available pre-death PA measurements to trajectory modelling and received trajectory assignments. No post-death measurements were imputed. Participants who died before T6 were excluded from the six-month cognitive outcome analysis.

Using participants without CRCI as the reference group, adjusted odds ratios and 95% confidence intervals were calculated to evaluate how significant predictive factors influenced the physical activity trajectory groups. Furthermore, subgroup analyses comparing changes in objective and subjective cognitive function between different physical activity groups were performed. Multiple imputation was performed with modified Multivariate Imputation by Chained Equations for the missing values [29]. All analyses were performed using STATA version 18 (StataCorp LLC). In particular, the *Traj* command was adopted to perform GBTM.

## Results

### Demographics and baseline information of study participants

Table 1 details the demographic information of the collected sample. This consisted of 327 patients enrolled between October 2023 and August 2025. A total of 54 patients withdrew from the study (attrition rate = 16.5%). Reasons included death (*n* = 32), unwilling to continue follow-up measures (*n* = 14), and loss of contact (*n* = 8). The proportion of male patients (58.1%) was slightly higher than that of females (41.9%). The mean age of the patients was 63.7 (standard deviation [SD]: 8.8) years. Most patients were newly diagnosed with stage III (26%) and IV NSCLC (34.6%). Of the total patients, 31.2% and 9.5% had hypertension and diabetes, respectively. Approximately 25.7% of the patients did not engage in moderate or high levels of physical activity, and the mean MET was 12.8 (SD: 19.9) hours per week.

**Table 1:** Baseline characteristics of study participants (*n* = 327)

	Mean (*SD*)		
	Overall	Male (*n* = 190)	Female (*n* = 137)
Age	63.7 (8.8)	63.9 (8.9)	63.4 (8.7)
Range	[40–82]	[40–82]	[43–82]
Duration of education, years	10.5 (3.9)	10.7 (3.8)	10.3 (4.1)
Stage of NSCLC, *n* (%)			
I	58 (17.7)	34 (17.9)	24 (17.5)
II	71 (21.7)	47 (24.7)	24 (17.5)
III	85 (26)	50 (26.3)	35 (25.5)
IV	113 (34.6)	59 (31.1)	54 (39.4)
Scheduled treatment at baseline, yes, *n* (%)	27 (8.3)	18 (9.5)	9 (6.6)
Surgery	2 (0.6)	2 (1.1)	0 (0)
Target therapy	8 (2.4)	6 (3.2)	2 (1.5)
Chemotherapy	20 (6.1)	12 (6.3)	8 (5.8)
Immunotherapy	1 (0.3)	1 (0.5)	0 (0)
Radiation therapy	8 (2.4)	5 (2.6)	3 (2.2)
History of disease/health condition, *n* (%)			
Hypertension	102 (31.2)	62 (32.6)	40 (29.2)
Diabetes	31 (9.5)	23 (12.1)	8 (5.8)
Congestive heart failure	1 (0.3)	0 (0)	1 (0.7)
Angina	2 (0.6)	2 (1.1)	0 (0)
Asthma	12 (3.7)	7 (3.7)	5 (3.6)
Kidney disease	3 (0.9)	1 (0.5)	2 (1.5)
Stroke	14 (4.3)	9 (4.7)	5 (3.6)
Arthritis	8 (2.4)	3 (1.6)	5 (3.6)
Objective cognitive function (HK-MoCA-5MIN)			
Memory	4.0 (1.1)	4.0 (1.1)	4.1 (1.1)
Language	7.1 (1.8)	6.9 (1.8)	7.3 (1.9)
Orientation	5.8 (0.6)	5.7 (0.6)	5.8 (0.5)
Delayed memory	7.4 (1.9)	7.3 (1.9)	7.7 (1.8)
Subjective cognitive function (FACT-Cog)			
CogPCI	54.2 (14.2)	53.7 (13.9)	54.8 (14.5)
CogQOL	13.9 (2.8)	13.9 (2.8)	14.1 (2.9)
CogOth	14.4 (3.6)	14.5 (3.6)	14.3 (3.6)
CogPCA	20.1 (5.0)	20.2 (5.1)	20.0 (4.9)
Cancer symptoms (MDASI)			
Symptom severity	2.3 (1.7)	2.4 (1.8)	2.1 (1.6)
Symptom interference	2.2 (2.4)	2.4 (2.5)	1.9 (2.1)
Self-efficacy for exercise (SEE)	42.6 (22.1)	41.4 (22.7)	44.2 (21.0)
Physical activity (IPAQ-SF), *n* (%)			
Low	84 (25.7)	52 (27.4)	32 (23.4)
Moderate	89 (27.2)	51 (26.8)	38 (27.7)
High	154 (47.1)	87 (45.8)	67 (48.9)
IPAQ-SF total MET, hours/week	12.8 (19.9)	12.4 (19.8)	13.5 (20.2)

*SD*, standard deviation; *NSCLC*, non-small cell lung cancer; *HK-MoCA-5MIN*, Montreal Cognitive Assessment 5-Minute Protocol Hong Kong Version; *FACT-Cog*, Functional Assessment of Cancer Therapy-Cognitive Function; *CogPCI*, perceived cognitive impairments; *CogQOL*, impact of perceived cognitive impairments on quality of life; *CogOth*, comments from others; *CogPCA*, perceived cognitive abilities; *MDASI*, MD Anderson Symptom Inventory; *SEE*, self-efficacy for exercise; *IPAQ-SF*, International Physical Activity Questionnaire–Short Form; *MET*, metabolic equivalent of task

### Prevalence of cancer-related cognitive impairment

Table 2 presents the point estimates of the CRCI prevalence at different timepoints, as assessed by the HK-MoCA 5MIN protocol. The prevalence of CRCI during the study observation period ranged from 17.5% to 29.1%. The prevalence of CRCI at the final follow-up timepoint (T6: 6 months after baseline measure) was 24.1% (95% CI: 19.2, 29.5).

**Table 2:** Prevalence of objective cancer-related cognitive impairment

			95% CI	
Timepoint	*n*	Prevalence (%)	Lower	Upper
Baseline	327	29.1	24.2	34.3
T1	300	26	21.1	31.4
T2	286	28	22.8	33.6
T3	277	25.3	20.3	30.8
T4	273	19	14.6	24.2
T5	274	17.5	13.2	22.5
T6	282	24.1	19.2	29.5

Objective cancer-related cognitive impairment as measured by the HK-MoCA

### Physical activity trajectory

**Fig. 1:**
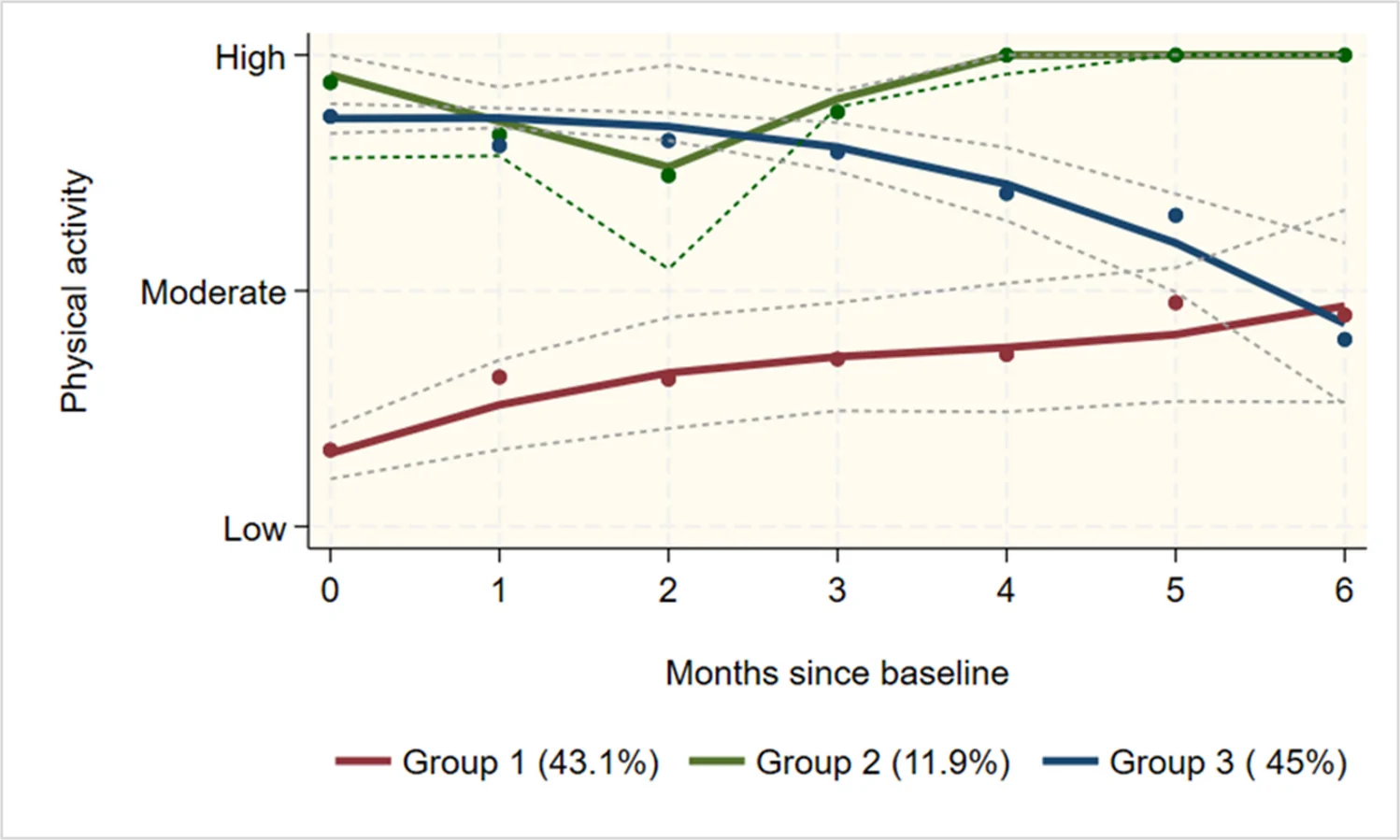
Physical activity trajectories: consistently low physical activity (group 1), consistently high physical activity (group 2), and decline (high to moderate) (group 3)

Based on criteria such as a smaller BIC, adequate group size, and clear interpretability, we selected a three-group model for further analysis. To visualize physical activity trajectories within the GBTM framework, we plotted the predicted average trajectory of physical activity across the study period (Fig. 1). As shown in Fig. 1, we identified three trajectories of the physical activity pattern: (1) consistently low physical activity (43.1%), (2) consistently high physical activity (11.9%), and (3) physical activity decline (high to moderate) (45%). Over the study period, the consistently low physical activity group maintained regular physical activity with slight increments but remained below a moderate level of physical activity. The decline group showed a quadratic decrease in their physical activity pattern. The consistently high physical activity group maintained a higher level of physical activity. These three trajectory groups started at a considerably different intercept at baseline. By the end of the study, the declining group engaged in less physical activity, whereas the consistently low physical activity group continued to increase their physical activity (Fig. 1).

Table 3 presents a comparison of physical activity patterns for study variables at the final follow-up. Throughout the period, the trajectories of the three groups significantly differed from each other at T6, with the consistent low and high physical activity groups exhibiting the lowest and highest HK-MoCA 5MIN Protocol scores on memory, language, and delay memory subdomains, respectively (*p* < 0.001; Table 3). Among the four subdomains of subjective cognitive function, the consistent high physical activity group outperformed the other two groups in perceived cognitive impairments (CogPCI: 62.9 [SD: 9.7]), impact of perceived cognitive impairments on quality of life (CogQOL: 15.5 [SD: 1.4]), comments from others (CogOth: 15.4 [SD:1.6]), and perceived cognitive abilities (CogPCA: 22.1 [SD: 5.7]), whereas the decline (high to moderate) group exhibited the lowest scores in perceived cognitive impairments (CogPCI: 51.3 [SD: 18.4]), comments from others (CogOth: 12.9 [SD: 4.5]), and perceived cognitive abilities (CogPCA: 18.2 [SD: 6.8]).

**Table 3:** Comparison of physical activity patterns for study variables at the final follow-up

	Physical activity patterns			
	Consistently low PA (*n* = 141)	Consistently high PA (*n* = 39)	PA decline (high to moderate) (*n* = 147)	*p*-value
	Mean (SD)	Mean (SD)	Mean (SD)	
Age	62.8 (9.9)	64.3 (6.7)	64.5 (7.9)	0.251
Female, *n* (%)	62 (44)	18 (46.2)	57 (38.8)	
FACT-Cog				
CogPCI	56.6 (14.2)	62.9 (9.7)	51.3 (18.4)	< 0.0001
CogQOL	12.6 (5.0)	15.5 (1.4)	12.6 (4.4)	0.001
CogOth	14.1 (3.2)	15.4 (1.6)	12.9 (4.5)	< 0.0001
CogPCA	21 (5.6)	22.1 (5.7)	18.2 (6.8)	< 0.0001
SEE	50.1 (19.6)	55.5 (17.6)	44.3 (26.2)	0.038
MDASI				
Symptom severity	1.6 (1.4)	0.8 (0.9)	2.6 (1.8)	< 0.0001
Symptom interference	1.3 (1.9)	0.6 (1.4)	3.1 (3.4)	< 0.0001
HK-MoCA 5MIN				
Memory	4.3 (1.0)	4.9 (0.2)	4.6 (0.6)	< 0.0001
Language	6.6 (2.3)	8.3 (1.1)	6.6 (1.7)	< 0.0001
Orientation	5.7 (0.5)	5.9 (0.2)	5.8 (0.5)	0.082
Delayed memory	8.5 (1.6)	9.7 (0.6)	8.9 (1.7)	< 0.0001
Cognitive impaired, n (%)	50 (38.8)	1 (2.6%)	17 (14.9)	< 0.0001

*SD*, standard deviation; *NSCLC*, non-small cell lung cancer; *HK-MoCA-5MIN*, Montreal Cognitive Assessment 5-Minute Protocol Hong Kong Version; *FACT-Cog*, Functional Assessment of Cancer Therapy-Cognitive Function; *CogPCI*, perceived cognitive impairments; *CogQOL*, impact of perceived cognitive impairments on quality of life; *CogOth*, comments from others; *CogPCA*, perceived cognitive abilities; *MDASI*, MD Anderson Symptom Inventory; *SEE*, self-efficacy for exercise; *PA*, physical activity

In addition to objective and subjective cognitive function, the decline (high to moderate) group reported the lowest self-efficacy for exercise (44.3 [SD: 26.2]) and the highest cancer symptom severity (2.6 [SD: 1.8]) and interference (3.1 [SD: 3.4]). The changes in objective and subjective cognitive function subdomains across the three physical activity pattern groups are presented in Supplementary Fig. S1 and S2.

### Predicting CRCI using physical activity trajectories

To investigate the effect of physical activity trajectories from baseline to T6 on cognitive impairment at T6, we conducted multivariate logistic regression (Table 4). Age, male sex, duration of education, advanced stage of NSCLC, and lower perceived cognitive impairments scores at baseline were identified as factors associated with CRCI, whereas self-efficacy for exercise was determined as a factor for CRCI (all *p* < 0.05, Table 4).

**Table 4:** Multivariate logistic regression for physical activity trajectories for cancer-related cognitive impairment

					95% Wald confidence limits	
Parameter	Beta	SE	*p*-value	OR	Lower	Upper
Age	−0.054	0.019	0.006	0.948	0.913	0.984
Female (ref: male)	−0.949	0.367	0.010	0.387	0.188	0.795
Duration of education	−0.246	0.050	< 0.0001	0.782	0.710	0.862
Stage of NSCLC: II (ref: Stage I)	−0.160	0.637	0.802	0.852	0.245	2.969
Stage of NSCLC: III (ref: Stage I)	1.599	0.586	0.006	4.950	1.570	15.607
Stage of NSCLC: IV (ref: Stage I)	1.079	0.563	0.055	2.940	0.975	8.866
FACT-Cog: CogPCI	−0.040	0.014	0.006	0.961	0.934	0.989
Self-efficacy for exercise	−0.017	0.008	0.034	0.983	0.968	0.999
Physical activity pattern						
Consistent high PA group (ref: consistent low PA group)	−2.784	1.061	0.009	0.062	0.008	0.494
PA decline (high to moderate) group (ref: consistent low PA group)	−1.955	0.423	< 0.0001	0.141	0.062	0.324

*OR*, odds ratio; *NSCLC*, non-small cell lung cancer; *FACT-Cog*, Functional Assessment of Cancer Therapy-Cognitive Function; *CogPCI*, perceived cognitive impairments; *PA*, physical activity

The available baseline information therefore did not characterize actual treatment exposure over the 6-month follow-up period, and treatment variables were not included in the adjusted regression model

After adjustment for potential covariates, the consistently high physical activity group had a 93.8% lower risk of CRCI (OR = 0.062, 95% CI 0.008, 0.494). Furthermore, the PA decline (high to moderate) group had an 85.9% (OR = 0.141, 95% CI 0.062, 0.324) lower risk of CRCI compared to those with consistently low physically activity group.

## Discussion

This prospective cohort study investigates the prevalence and predictors of CRCI, and the trajectory of physical activity patterns, in patients newly diagnosed with NSCLC in Hong Kong. The results contribute to the scarce literature on cognitive health in lung cancer survivorship and emphasize the association with cognitive outcomes of physical activity patterns in modulating CRCI risk over a six-month follow-up period.

### Prevalence of CRCI in newly diagnosed lung cancer patients

The observed prevalence of objective CRCI ranged from 17.5% to 29.1% across measurement points, with 24.1% affected at six months post-diagnosis. This prevalence mirrors previous studies in lung cancer and broader oncology populations [5], stressing that CRCI is a common and clinically important survivorship challenge. The temporal variability suggests cognitive function may fluctuate during the cancer trajectory, possibly influenced by treatment phases, symptom burden, and psychosocial stressors [4, 8]. Hence, reliance on cognitive screening at a single time-point may underestimate or overlook patients developing later cognitive impairment, particularly perceived cognitive impairment. Our team has developed an ecological momentary mobile cognitive screening for CRCI among patients with lung cancer, which appears to be a feasible solution to monitoring the fluctuations of cognitive functioning change over time [30]. Through assessing the fluctuation of cognitive function changes, the cognitive variability can be captured. Further research is warranted to investigate cognitive variability as a predictor of cancer patients’ cognitive health. This supports clinical guidelines recommending longitudinal cognitive monitoring in cancer care to identify and manage emerging deficits [6].

### Physical activity trajectories: profiles and cognitive function correlates

A key novel insight is the characterization of three distinct physical activity trajectories: consistently low, consistently high, and declining from high to moderate activity. This differentiation extends understanding beyond binary active/inactive classifications to a nuanced depiction of how physical activity behavior changes in lung cancer patients. Notably, both consistently high physical activity and declining physical activity trajectories showed substantially decreased CRCI risk compared with persistently low physical activity. Mechanistically, physical activity may attenuate CRCI by enhancing neuroplasticity, promoting cerebral perfusion, reducing systemic inflammation, and mitigating neurotoxic effects of cancer and treatments [31]. The marked 93.8% risk reduction in the consistently high physical activity group aligns with the literature, demonstrating exercise’s neuroprotective effects in cancer survivors and aging populations [32]. The declining physical activity group’s robustness in cognitive outcomes, despite a decrease to moderate activity, suggests that even moderate physical activity levels may suffice for cognitive preservation or that early high activity confers lasting benefits [15, 16].

Clinically, these results emphasize the importance of early identification and sustained promotion of physical activity in lung cancer care. The decline trajectory may reflect patients whose symptom burden or psychological distress reduces activity over time, indicating a vulnerable group in need of tailored interventions [33]. Encouraging maintenance of physical activity through symptom management, motivational support, and adaptive exercise programmes could mitigate CRCI development or progression.

### Cancer symptoms and its impact on physical activity and cognition

The highest symptom severity and interference scores in the declining physical activity group highlight the negative interplay between symptom burden and physical activity engagement. Symptoms such as fatigue, pain, dyspnea, and mood disorders common in lung cancer patients can limit their physical capabilities and motivation, creating a cyclical scenario where inactivity exacerbates both symptomatology and cognitive impairment [33]. Furthermore, low self-efficacy for exercise in the declining physical activity group indicates psychosocial barriers that may hinder physical activity engagement. Exacerbation of cognitive impairment under these conditions demonstrates the need for integrated supportive care encompassing symptom control, psychological support, and behavioral interventions to boost exercise confidence [34]. Clinicians should prioritize comprehensive symptom assessment and management alongside motivational strategies and education on the cognitive and functional benefits of exercise.

### Objective and subjective cognitive function

The study’s combined use of objective (HK-MoCA 5MIN) and subjective (FACT-Cog) measures provides a holistic view of CRCI. The consistent high physical activity group’s superior performance in several domains, including memory, language, delay memory, perceived cognitive impairments, and quality of life impact, validates the multifaceted association with cognitive outcomes of physical activity.

Interestingly, subjective cognitive complaints often reflect emotional distress or functional impact beyond measurable neuropsychological deficits and predict future cognitive decline [35]. Their misalignment with objective findings in this study points to the clinical utility of including patients’ perspectives in CRCI evaluation. It also supports tailored psychosocial interventions addressing both perceived and actual impairments [36]. In our subgroup analysis, patients in the consistently low physical activity group showed higher scores than the decline (high to moderate) group in each subjective cognitive function subdomain over time, where the between-group differences in objective cognitive function subdomains were not obvious. These results further support that subjective and objective cognitive functions are poorly correlated [37].

### Other predictors of CRCI

Male sex, lower education, advanced NSCLC stage, and lower baseline perceived cognitive function emerged as significant CRCI predictors. These align with existing literature linking diminished cognitive reserve (via lower education) and cancer severity to increased neurotoxicity risk [38]. The sex difference observed warrants further investigation, as evidence on gender and CRCI remains mixed, potentially influenced by hormonal, genetic, or psychosocial factors. Self-efficacy for exercise’s role underscores the behavioral and psychological components influencing CRCI, suggesting that enhancing patients’ confidence in managing physical activity may indirectly support cognitive health.

### Implications for clinical practice

Routine CRCI screening using validated, brief instruments like HK-MoCA 5MIN at multiple post-diagnosis time points should be integrated into lung cancer care if the routine use of more comprehensive neuropsychological tests is not feasible in clinical practice. Efforts to promote physical activity must prioritize tailored approaches accounting for patients’ symptom severity, functional status, motivation, and psychosocial context [39]. Multidisciplinary collaboration among oncologists, rehabilitation and exercise specialists, psychologists, and nursing staff are vital to designing and delivering comprehensive support encompassing exercise prescription, symptom management, and self-efficacy enhancement [40]. This study highlights key research priorities. Randomized controlled trials of tailored exercise and cognitive rehabilitation are needed to establish causality and determine the optimal timing, intensity, and type of physical activity. Neurobiological studies using imaging, biomarkers, and genetics can clarify CRCI mechanisms and activity-related protection, while long-term follow-up studies will define the persistence of CRCI and lasting benefits of sustained activity.

### Strengths and limitations

This study’s strengths include its prospective design, longitudinal repeated assessments, use of validated cognitive and physical activity instruments, and advanced trajectory modelling allowing detailed physical activity pattern characterization. Its focus on lung cancer addresses an underexplored and high-risk group for CRCI. Several limitations need to be acknowledged. First, we did not include potential confounders such as depression, anxiety, or genetic predisposition, which may independently affect perceived cognition. Physical activity trajectories may partly reflect underlying health status, including disease severity, symptom burden, and functional capacity, rather than an independent protective effect of physical activity. Although our analyses adjusted for selected demographic and clinical characteristics, residual confounding remains possible. Furthermore, physical activity and cognitive function were assessed over overlapping periods, so reverse causation cannot be excluded. The observed associations should therefore not be interpreted as evidence that increasing physical activity independently reduces CRCI. In addition, actual treatment exposure during follow-up was not assessed. Baseline treatment scheduling could not adequately capture treatment initiation, duration, or potentially concurrent and sequential modalities. Treatment variables were therefore not included in the adjusted model, and residual treatment-related confounding remains possible. Second, although attrition was modest, loss to follow-up over 6 months may bias results. Mortality-related attrition may have introduced survivor selection. Although deceased participants contributed their available pre-death PA measurements to trajectory modelling, they could not contribute 6-month cognitive outcomes. If these participants had greater cognitive vulnerability than survivors, their absence may have led to underestimation of cognitive impairment in the baseline cohort. Mortality-related selection may also have affected the observed associations between PA trajectories and cognition, although the magnitude and direction of this bias are uncertain. Third, a convenience sampling was employed which may limit the generalisability and external validity. Fourth, the MoCA is essentially a screening test for mild cognitive impairment and is not a comprehensive neuropsychological battery. It is likely to underestimate CRCI as it is less likely to detect the more subtle cognitive impairment commonly seen in cancer patients.

Our study demonstrates that CRCI affects a subset of NSCLC patients within 6 months of diagnosis, with physical activity patterns closely linked to cognitive outcomes. Regular activity offers neuroprotective benefits, whereas high symptom burden and low exercise self-efficacy impair cognition by reducing activity. Incorporating CRCI screening, symptom management, and tailored exercise promotion into lung cancer care can improve survivors’ quality of life.

## Supplementary Information

Below is the link to the electronic supplementary material.

ESM 1 DOCX (72.0 KB)

## Data Availability

The data that support the findings of this study are available from the author [MHH] upon reasonable request.
