# Supplementary material for: Prevalence, predictive factors, and the influential role of physical activity trajectories of cancer-related cognitive impairment among patients newly diagnosed with non-small cell lung cancer
Source: Support Care Cancer. 2026 Oct 24;34(11):1152. doi: 10.1007/s00520-026-11368-5 (PMC13695631; doi:10.1007/s00520-026-11368-5)
Supplement: Supplementary file 1 [file 520_2026_11368_MOESM1_ESM.docx]

| **Objective cognitive function subdomains** | |
| --- | --- |
| (A) | (B) |
|  |  |
| (C) | (D) |
|  |  |

Memory

|  | Group 1 | Group 2 | Group 3 |
| --- | --- | --- | --- |
| Baseline | 3.93 | 4.72 | 4.05 |
| T1 | 3.91 | 4.74 | 4.31 |
| T2 | 4.29 | 4.82 | 4.45 |
| T3 | 4.09 | 4.68 | 4.18 |
| T4 | 4.4 | 4.58 | 4.5 |
| T5 | 4.26 | 4.89 | 4.72 |
| T6 | 4.29 | 4.95 | 4.6 |

Language

|  | Group 1 | Group 2 | Group 3 |
| --- | --- | --- | --- |
| Baseline | 7.35 | 7.67 | 6.66 |
| T1 | 7.81 | 7.42 | 6.74 |
| T2 | 7.06 | 7.27 | 6.92 |
| T3 | 7.27 | 7.82 | 7.21 |
| T4 | 7.08 | 7.51 | 6.44 |
| T5 | 7.02 | 8.08 | 6.53 |
| T6 | 6.75 | 8.31 | 6.57 |

Orientation

|  | Group 1 | Group 2 | Group 3 |
| --- | --- | --- | --- |
| Baseline | 5.78 | 5.74 | 5.82 |
| T1 | 5.91 | 5.85 | 5.78 |
| T2 | 5.79 | 6 | 5.77 |
| T3 | 5.87 | 5.97 | 5.8 |
| T4 | 5.87 | 5.94 | 5.86 |
| T5 | 5.78 | 5.92 | 5.84 |
| T6 | 5.75 | 5.95 | 5.75 |

Delayed Memory

|  | Group 1 | Group 2 | Group 3 |
| --- | --- | --- | --- |
| Baseline | 7.48 | 8.56 | 7.11 |
| T1 | 7.63 | 8.38 | 8.14 |
| T2 | 8.17 | 9.31 | 8.1 |
| T3 | 8.5 | 8.84 | 8.34 |
| T4 | 8.38 | 8.92 | 8.71 |
| T5 | 8.71 | 9.44 | 8.69 |
| T6 | 8.59 | 9.77 | 8.87 |

**Supplementary Figure S1.** The changes in objective and subjective cognitive function subdomains between three physical activity pattern groups

***Note.*** Physical activity trajectories: consistently low physical activity (group 1), consistently high physical activity (group 2), and decline (high to moderate) (group 3).

| **Subjective cognitive function subdomains** | |
| --- | --- |
| (E) | (F) |
|  |  |
| (G) | (H) |
|  |  |

CogPCI

|  | Group 1 | Group 2 | Group 3 |
| --- | --- | --- | --- |
| Baseline | 56.11 | 61.66 | 50.34 |
| T1 | 57.42 | 60.71 | 52.68 |
| T2 | 58.88 | 63.79 | 56.31 |
| T3 | 58.35 | 63.94 | 54.8 |
| T4 | 60.21 | 64 | 49.65 |
| T5 | 58.79 | 63.75 | 56.89 |
| T6 | 56.64 | 62.97 | 51.35 |

CogQOL

|  | Group 1 | Group 2 | Group 3 |
| --- | --- | --- | --- |
| Baseline | 14.1 | 15.69 | 13.42 |
| T1 | 13.45 | 14.74 | 12.61 |
| T2 | 14 | 15.1 | 13.65 |
| T3 | 14.54 | 15.6 | 12.64 |
| T4 | 14.87 | 14.8 | 11.15 |
| T5 | 14.28 | 15.52 | 13.13 |
| T6 | 12.65 | 15.48 | 12.57 |

CogOth

|  | Group 1 | Group 2 | Group 3 |
| --- | --- | --- | --- |
| Baseline | 14.93 | 15.28 | 13.63 |
| T1 | 14.23 | 14.87 | 14.79 |
| T2 | 15.31 | 14.84 | 14.66 |
| T3 | 14.39 | 15.13 | 13.58 |
| T4 | 14.9 | 15.13 | 14.54 |
| T5 | 14.17 | 15.11 | 14.17 |
| T6 | 14.07 | 15.48 | 12.94 |

**Supplementary Figure S2.** The changes in objective and subjective cognitive function subdomains between three physical activity pattern groups

***Note.*** Physical activity trajectories: consistently low physical activity (group 1), consistently high physical activity (group 2), and decline (high to moderate) (group 3).

**Abbreviations:** CogPCI, perceived cognitive impairments; CogQOL, impact of perceived cognitive impairments on quality of life; CogOth, comments from others; CogPCA, perceived cognitive abilities.
